# Supplementary figures and images for: Data-driven segmentation of cortical calcium dynamics
Source: PLoS Comput Biol. 2023 May 1;19(5):e1011085. doi: 10.1371/journal.pcbi.1011085 (PMC10174627; doi:10.1371/journal.pcbi.1011085)

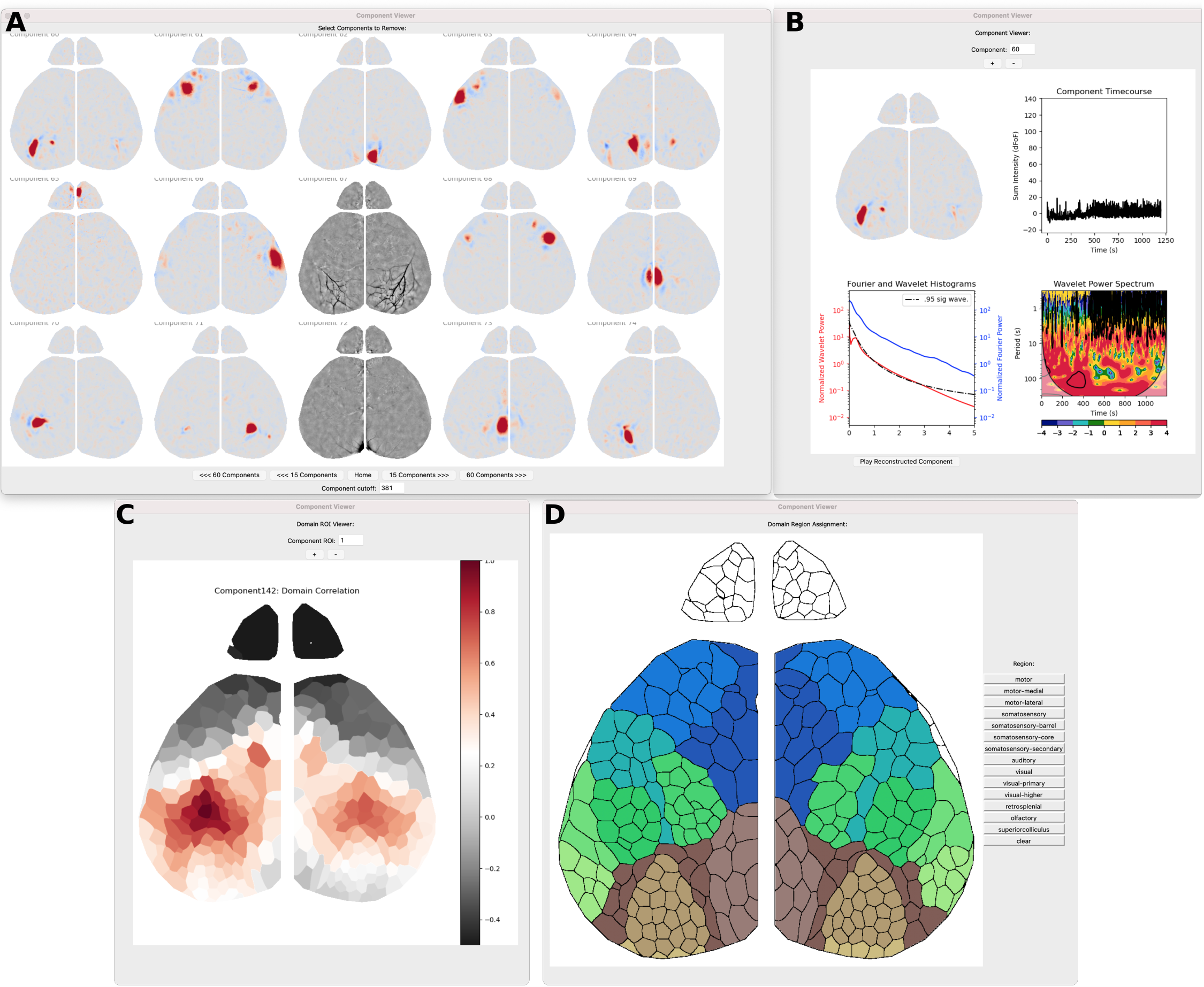

Supplement: S1 Fig — (A) 15 independent components, order 60–74 by variance. Components displayed in gray are manually selected as artifact either manually or using a machine learning classifier. A click on the display for any given component manually toggles its classification as either signal or artifact associated. Components colored in the cool/warm colormap are neural associated. Components colored in the black/white colormap are artifact associated. Buttons on the bottom panel control GUI movement through the dataset. The text panel at the bottom displays the index used as the signal/noise cutoff. (B) The component viewer displays additional temporal metrics for any given component. The top controls allow movement through the dataset by manual scrolling with (+/-) buttons, up/down keys, or through typing a desired component in the text box. sIC time course displays the mixing matrix time course extracted by ICA for the given components. The Wavelet power spectrum is displayed in the bottom right, and an integrated wavelet or Fourier representation is available on the bottom left. 0.95 significance as estimated by the AR(1) autoregressive red-noise null hypothesis is displayed as a dot-dash line. (C) The domain map correlation page shows the Pearson’s correlation coefficient between a selected seed domain and every other domain detected on the cortical surface. The seed domain can be changed through the arrow keys, the (+/-) buttons, or by clicking on a different domain on the displayed domain map. (D) The Component region assignment page allows manual region assignment for each domain. After the region is selected from the menu on the right, each domain clicked on the domain map is assigned to that region. (TIF) [file pcbi.1011085.s001.tif]

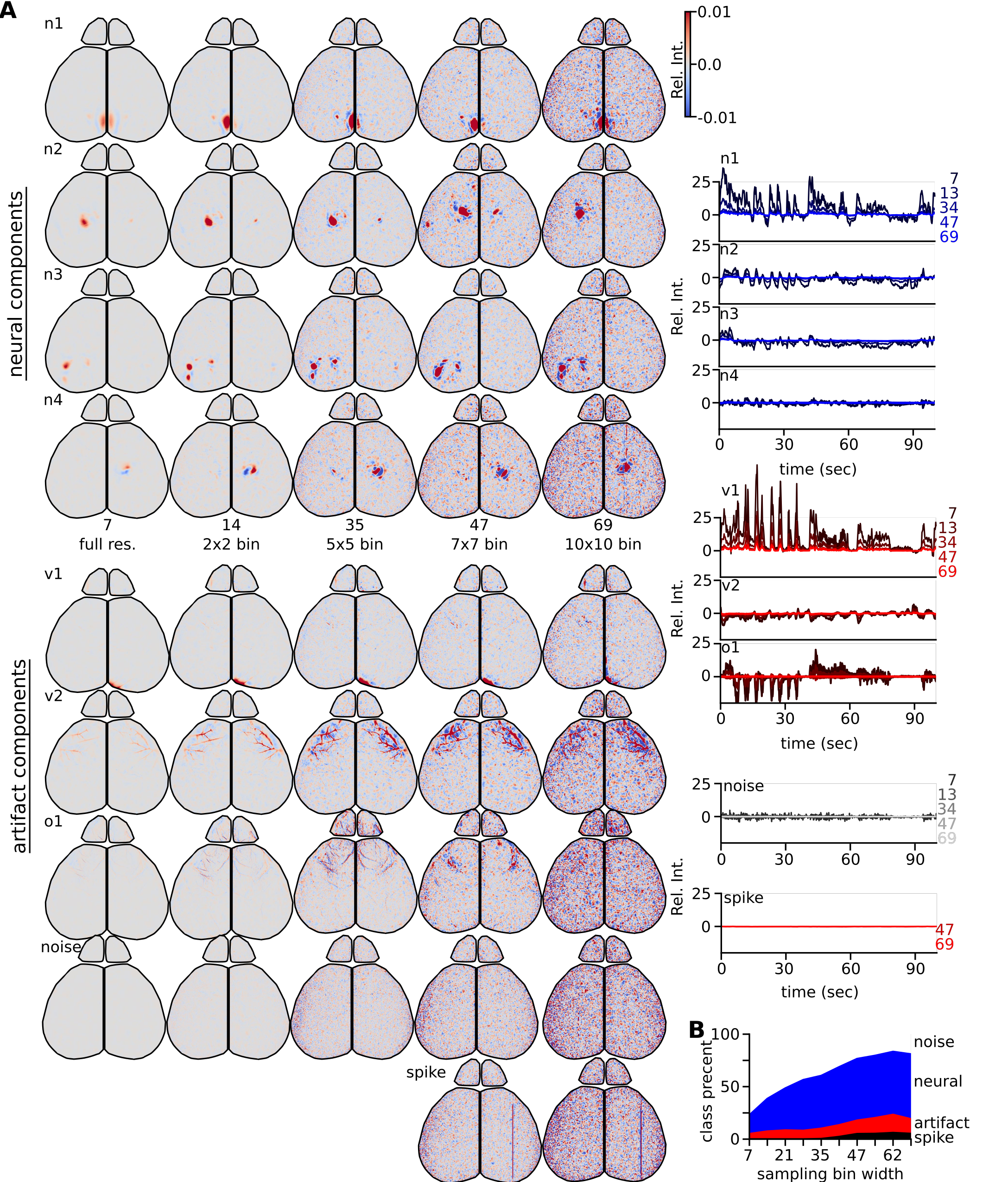

Supplement: S2 Fig — (A) Comparable examples of neuronal (top) and artifact (bottom) sIC (left) with their corresponding time series plotted on top of each other (right). Moving left to right shows a decrease in the spatial sampling rate of the decomposition. (B) Emergence of spike-like sICs were seen after 35um down sampling. (TIF) [file pcbi.1011085.s002.tif]

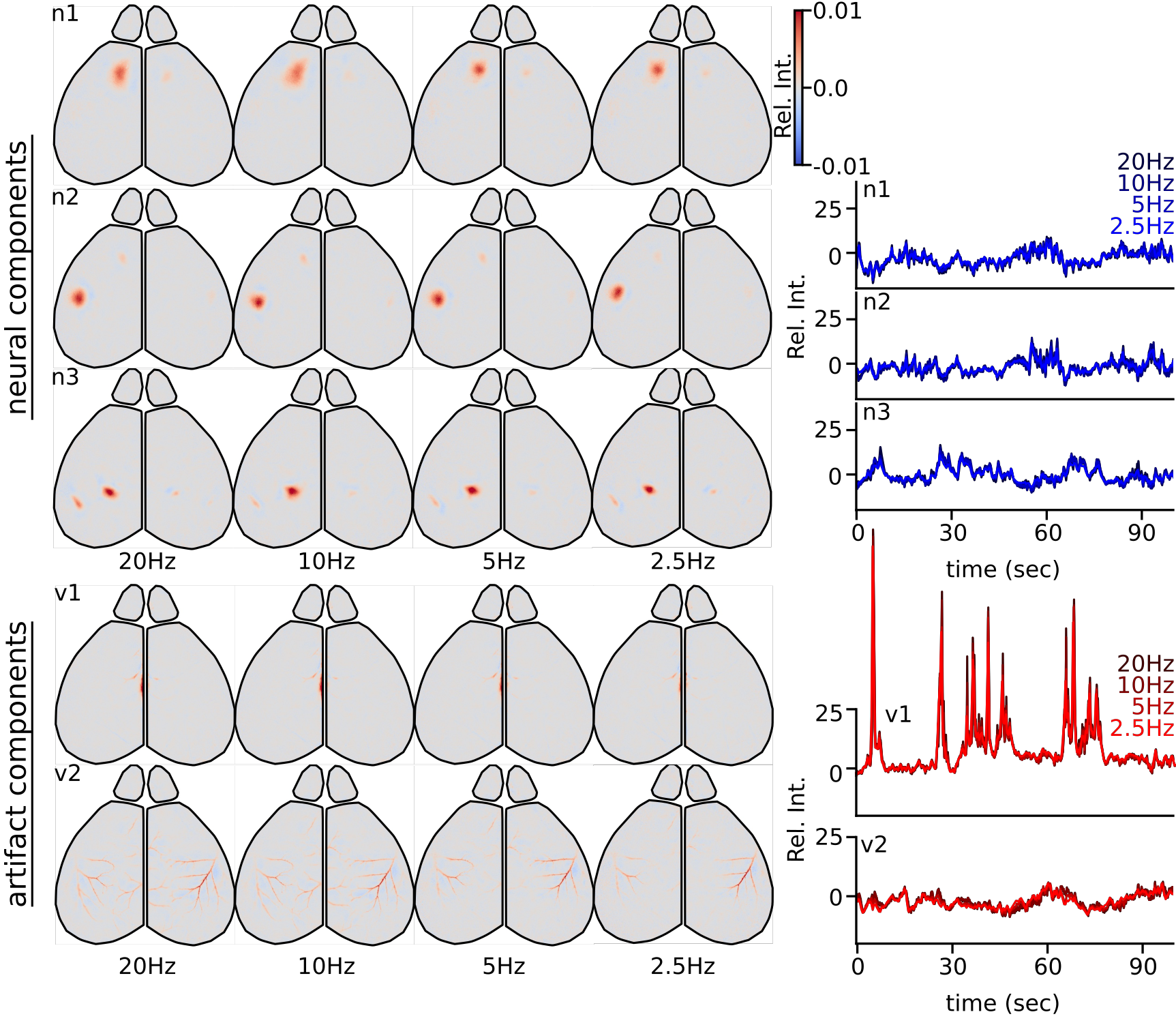

Supplement: S3 Fig — Comparable examples of neuronal (top) and artifact (bottom) sIC (left) with their corresponding time series plotted on top of each other (right). Moving left to right shows a decrease in the temporal sampling rate of the decomposition. (TIF) [file pcbi.1011085.s003.tif]

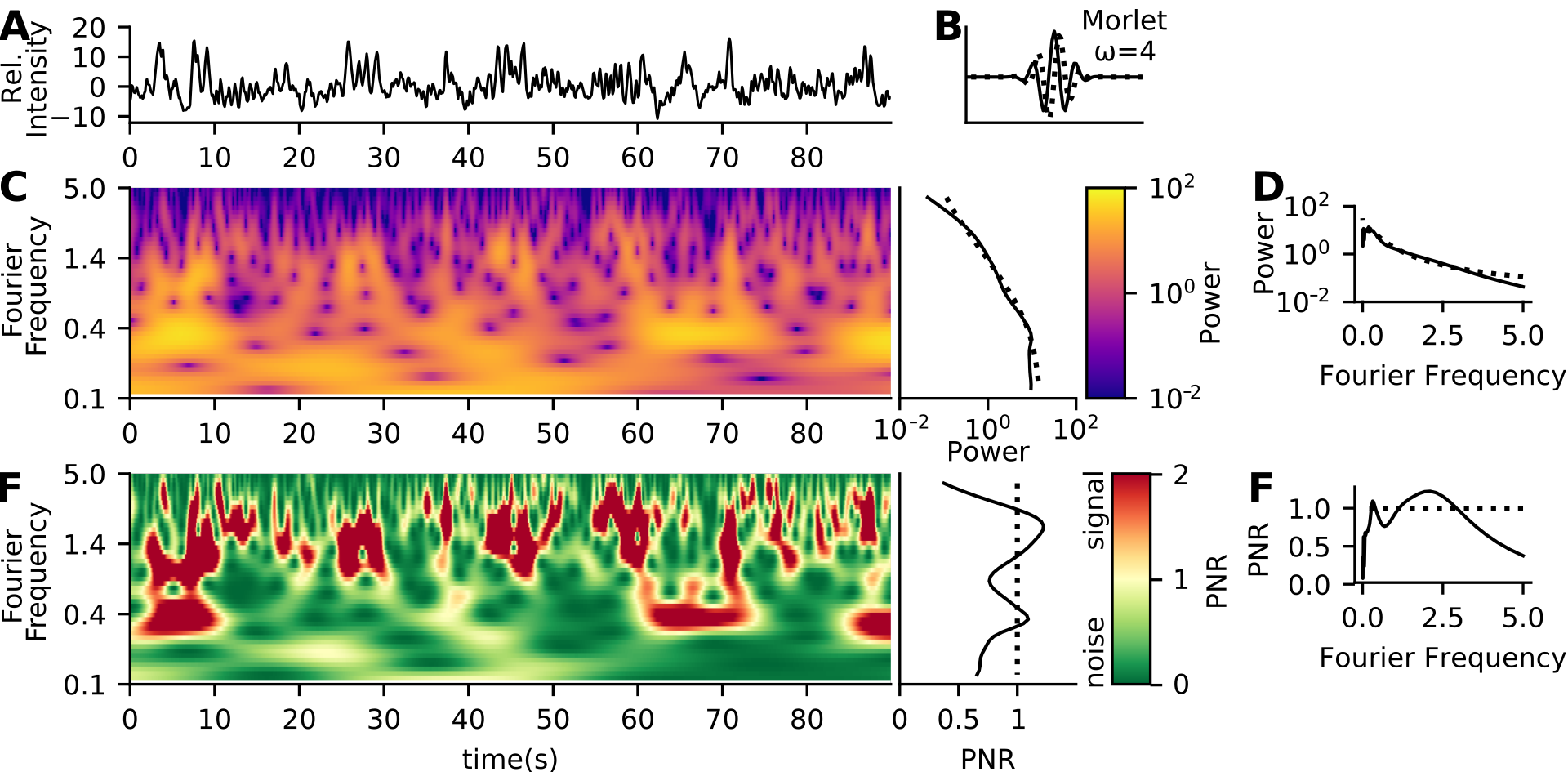

Supplement: S4 Fig — (A) Example neural time series, 90 sec of data recorded at 10hz reported in the temporal portion of a component (B) Morlet wavelet (ω = 4) was used for the wavelet transform. (C) The power spectra of the wavelet transform (colorbar, blue to yellow) and the global spectral analysis (black, right). The 95% quantile is shown in dashed lines on the global spectral analysis. Reformatting the frequency spacing, produces. (D) A power signal-to-noise ratio is calculated by dividing the power spectra by the 95% quantile of red noise defined by the AR(1). All values above 1 (dashed line) would indicate a high probability of signal. Anything below 1 would most likely be considered noise (colorbar, green to red). (F) Reformatted frequency spacing. (TIF) [file pcbi.1011085.s004.tif]

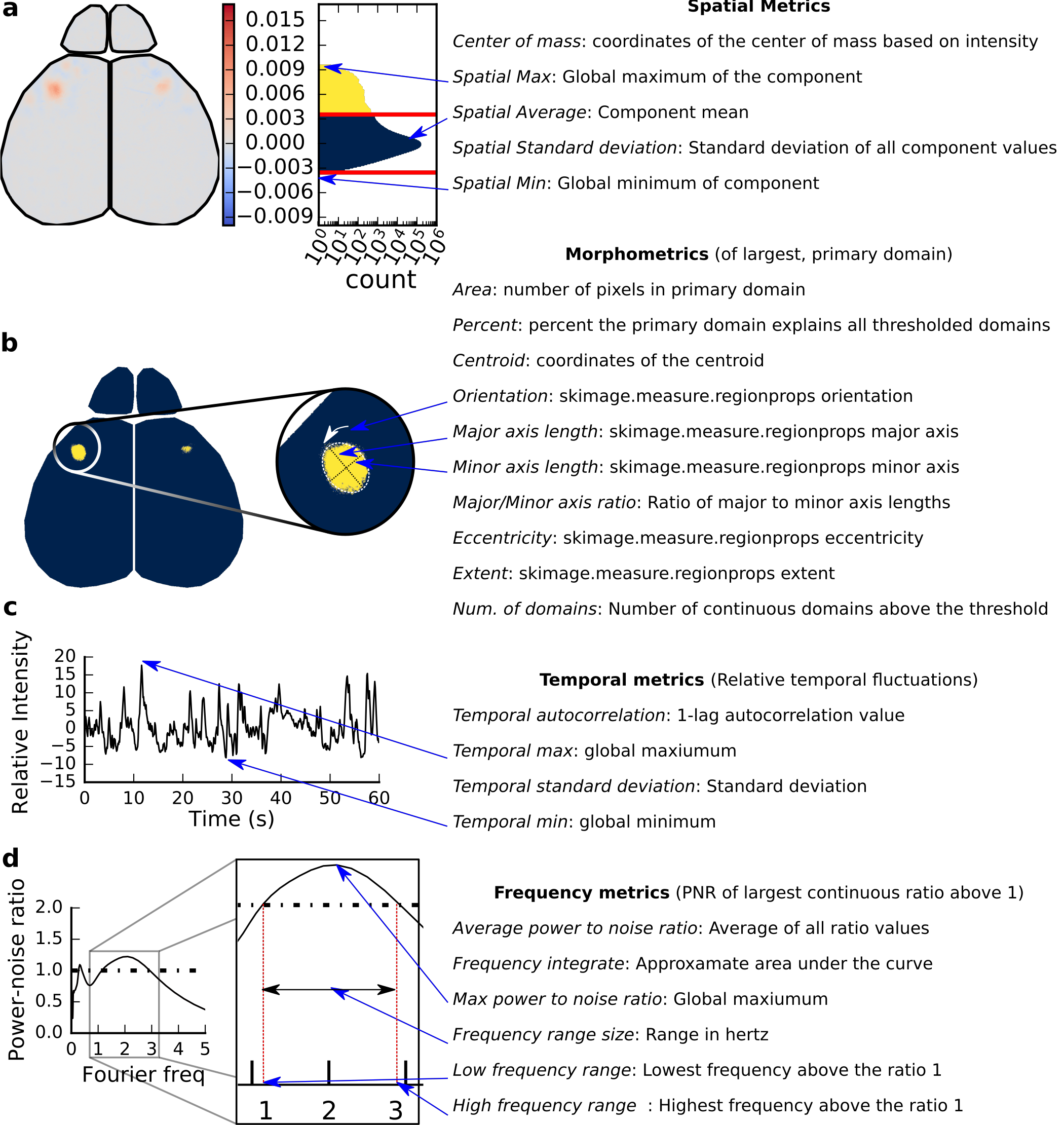

Supplement: S5 Fig — (A) Spatial metrics from statistical characteristics of each sIC (spatial representation of the component). The histogram of all sIC values is shown the right of the sIC. (B) Morphometrics collected from the binarized thresholded masked region of the sIC. The largest (primary) domain was used to generate the features for each sIC. The majority of metrics calculated utilizes sci-kit image region properties. (C) Temporal metrics are statistical descriptors from the corresponding row of the mixing matrix for each sIC. (D) Frequency analysis was done on the mixing matrix row, utilizing the PNR calculated from wavelet analysis (S4 Fig). The longest of all continuous frequencies was used to extract each feature. (TIF) [file pcbi.1011085.s005.tif]

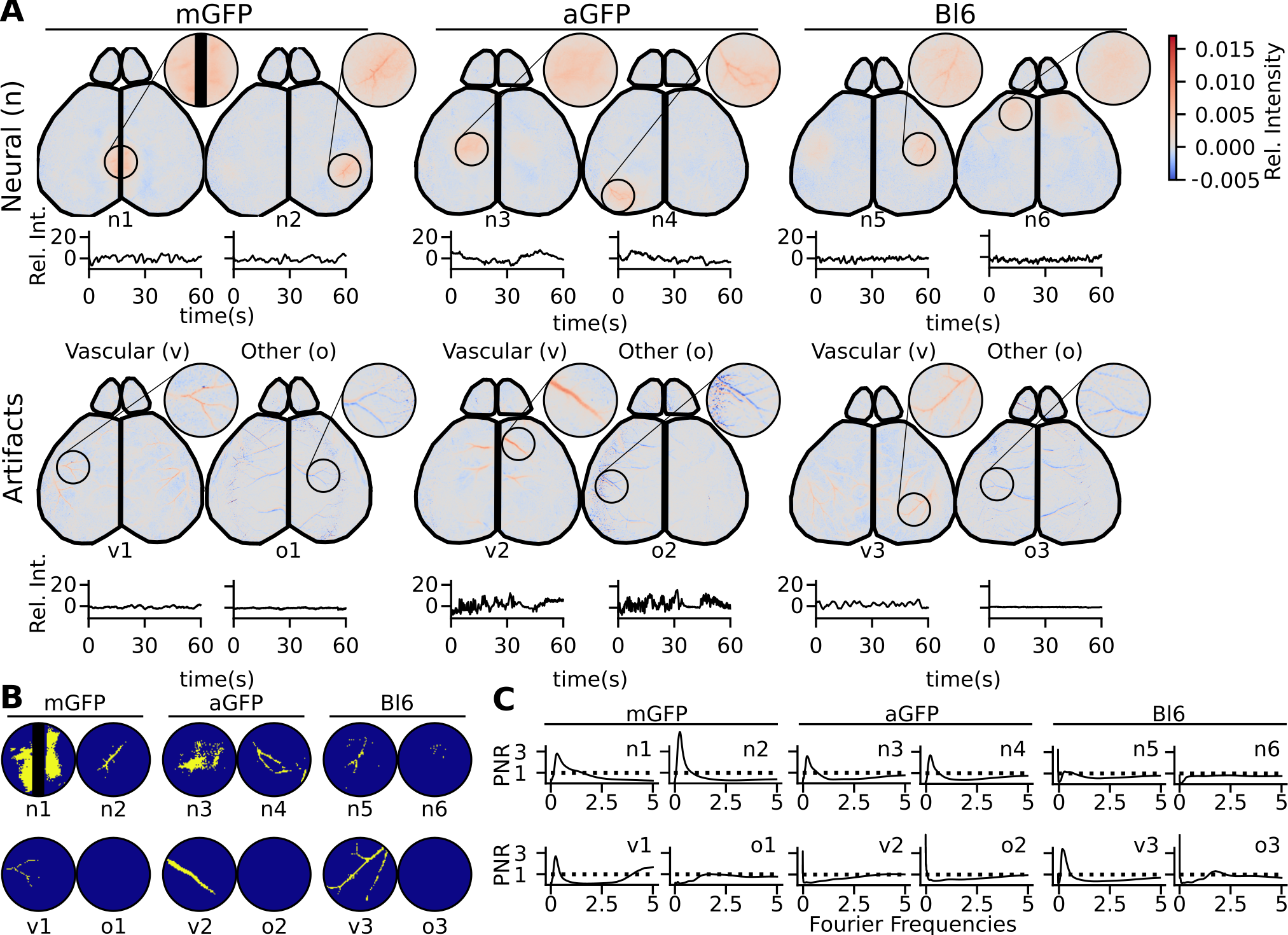

Supplement: S6 Fig — (A) Control components from 20 minutes of recording from cx3cr1 GFP (microglia; mGFP, left), adlh1 GFP (astrocyte; aGFP, center), and Black 6 (Non-transgenic; Bl6, right) mice. Two IC examples from each control group corresponding to hemodynamics/neural activity (top) and artifacts (bottom). Artifacts chosen show a vascular and other artifact commonly seen in GCaMP recordings. Similar data description in regards to temporal and spatial representations as seen in Fig 1. (B) Examples of control binarization of the sIC only showing the windowed spatial representation on the key portions of the sIC. (C) Examples of neural and artifact wavelet analysis shown in the power signal-to-noise ratio (PNR) plots. (TIF) [file pcbi.1011085.s006.tif]

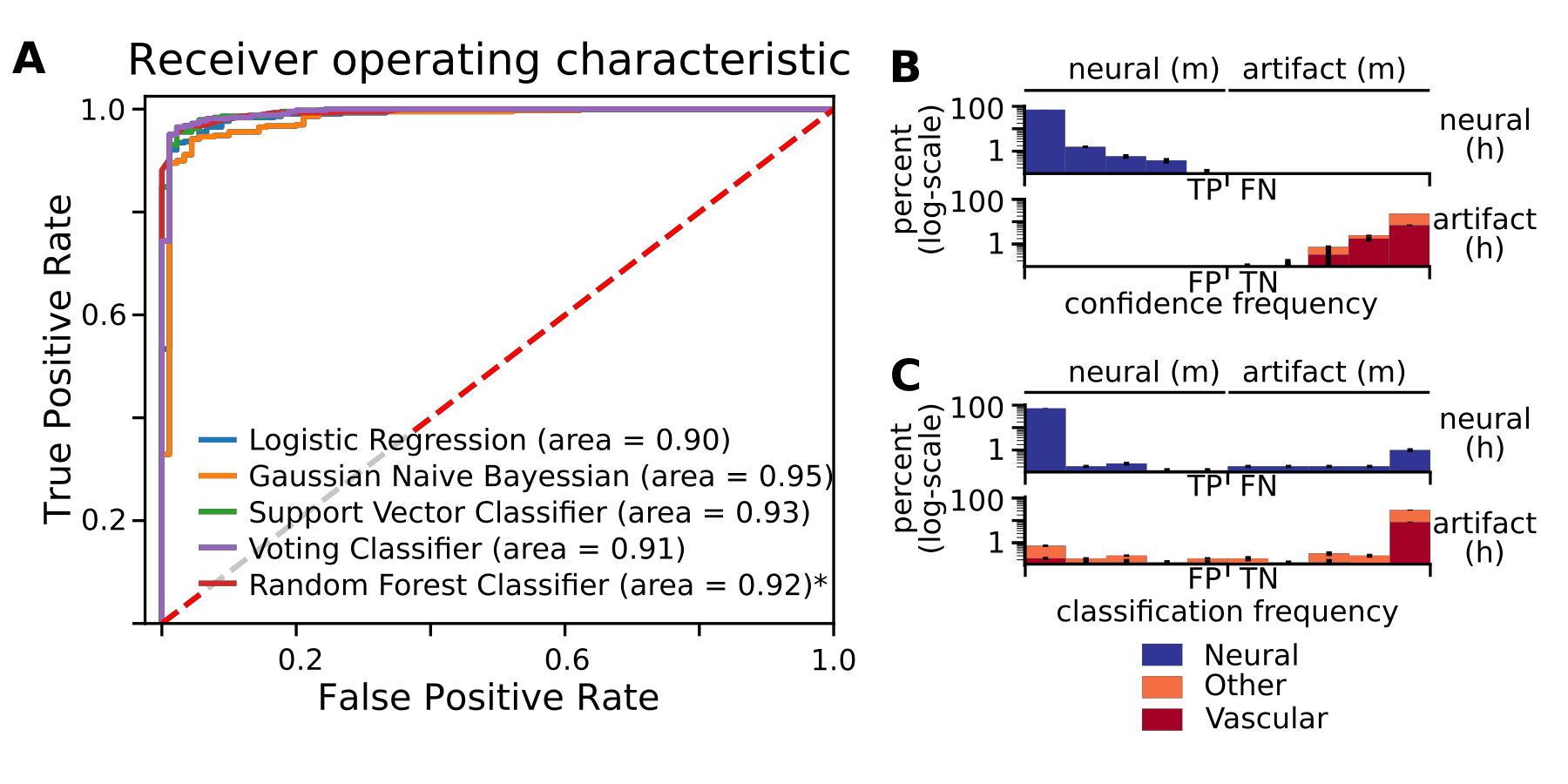

Supplement: S7 Fig — A) Receiver operating characteristic (ROC) plots for each classifying algorithm utilized. Voting classifier was composed of the 4 other algorithms. Random Forest Classifier (RFC; *) was used in all analyses in this paper. B) Histogram of human classification with the percent and error of each training iteration, binned based on confidence (same data as Fig 6F, bottom). Log-scale was used to highlight the low percentage points. C) Histogram of human classification with the percent in each binned novel classification. Classification bins based on the percent each classification occurred correctly in the 1000 trials. True positive (TP), False positive (FP), False negative (FN), True Negative (TN), human (h), machine (m). (TIF) [file pcbi.1011085.s007.tif]

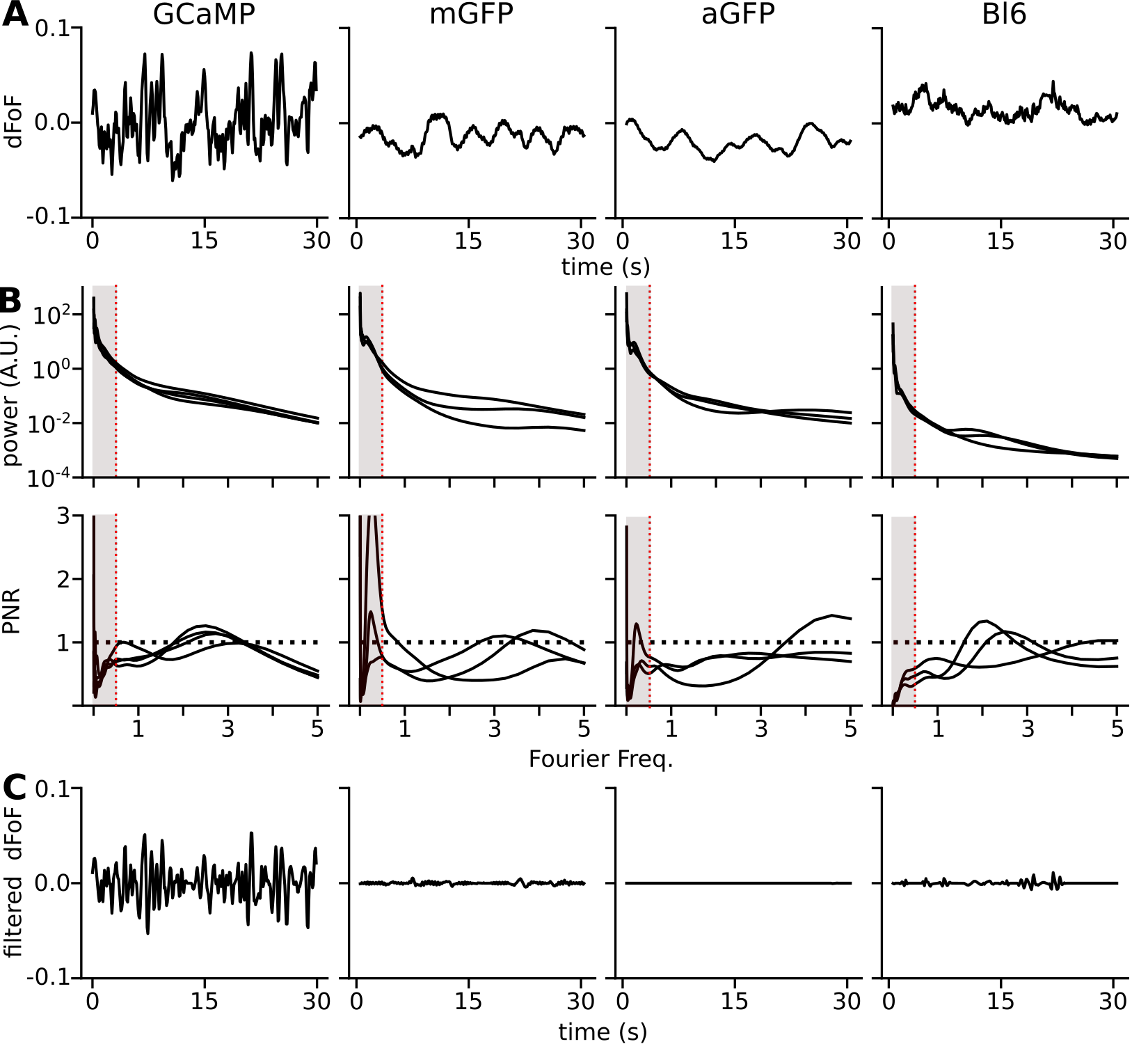

Supplement: S8 Fig — (A) 30sec examples of the global mean that was subtracted and stored at the initiation of the pipeline, prior to before the decomposition into sIC for GCaMP, mGFP, aGFP and Bl6. (B) Global wavelet spectrum (top) and its corresponding power to noise ratio (PNR; bottom) of GCaMP (N = 4), mGFP (N = 3), aGFP (N = 3), and Bl6 (N = 3), red indicates the omitted frequencies from our applied high pass filter. (C) High-pass filtration results of the same 30 sec in A. (TIF) [file pcbi.1011085.s008.tif]

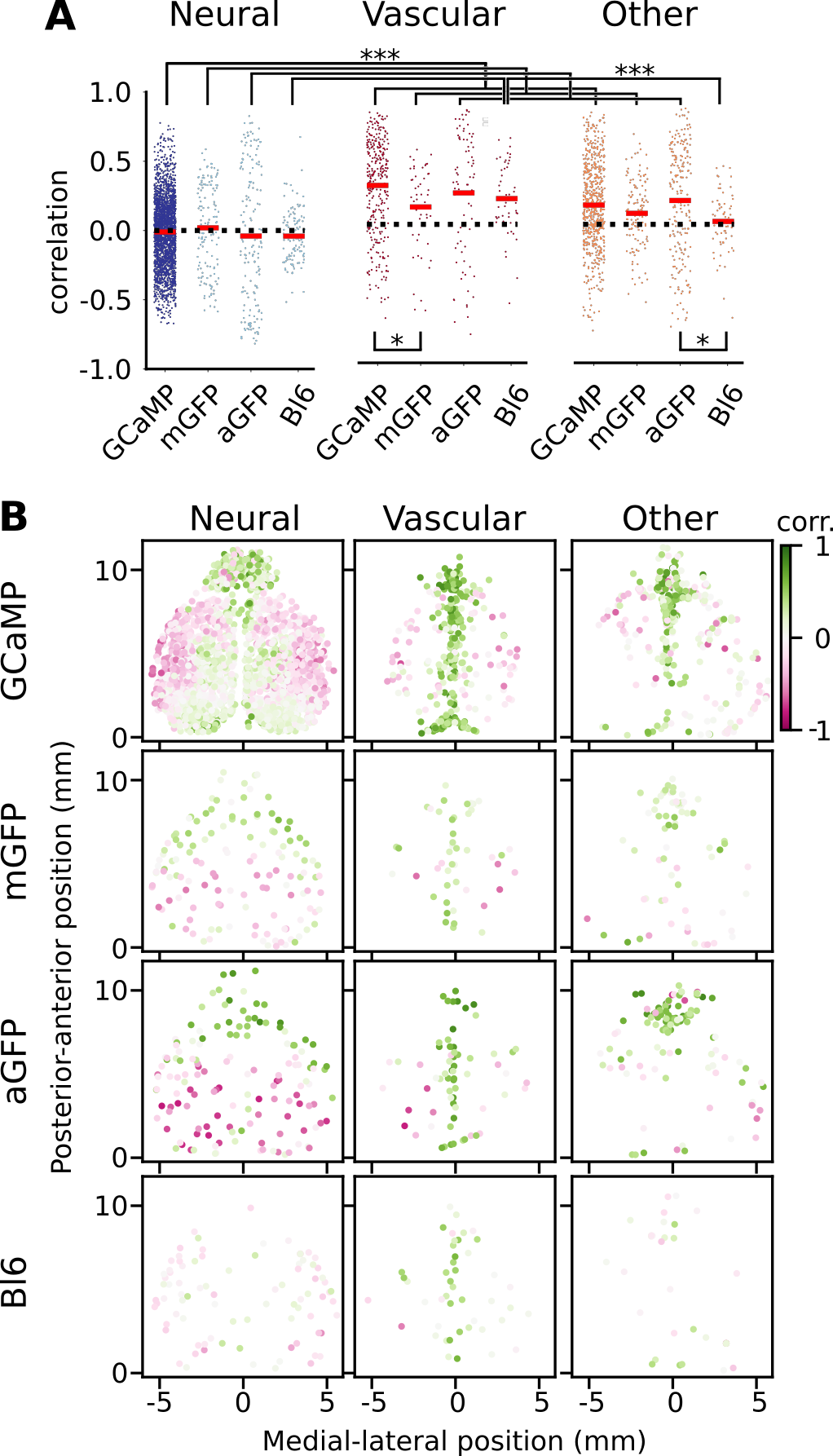

Supplement: S9 Fig — (A) All neural (blue), vascular (red), and other (orange) components and their correlation to the motion vector from each animal. (B) Spatial location and corresponding correlation (green to pink) of each component to motion based on their respective classification and genetic background. neural: left column, vascular: center column, other: right column. Top row: GCaMP, second row: mGFP, third row: aGFP, last row: Bl6. (TIF) [file pcbi.1011085.s009.tif]

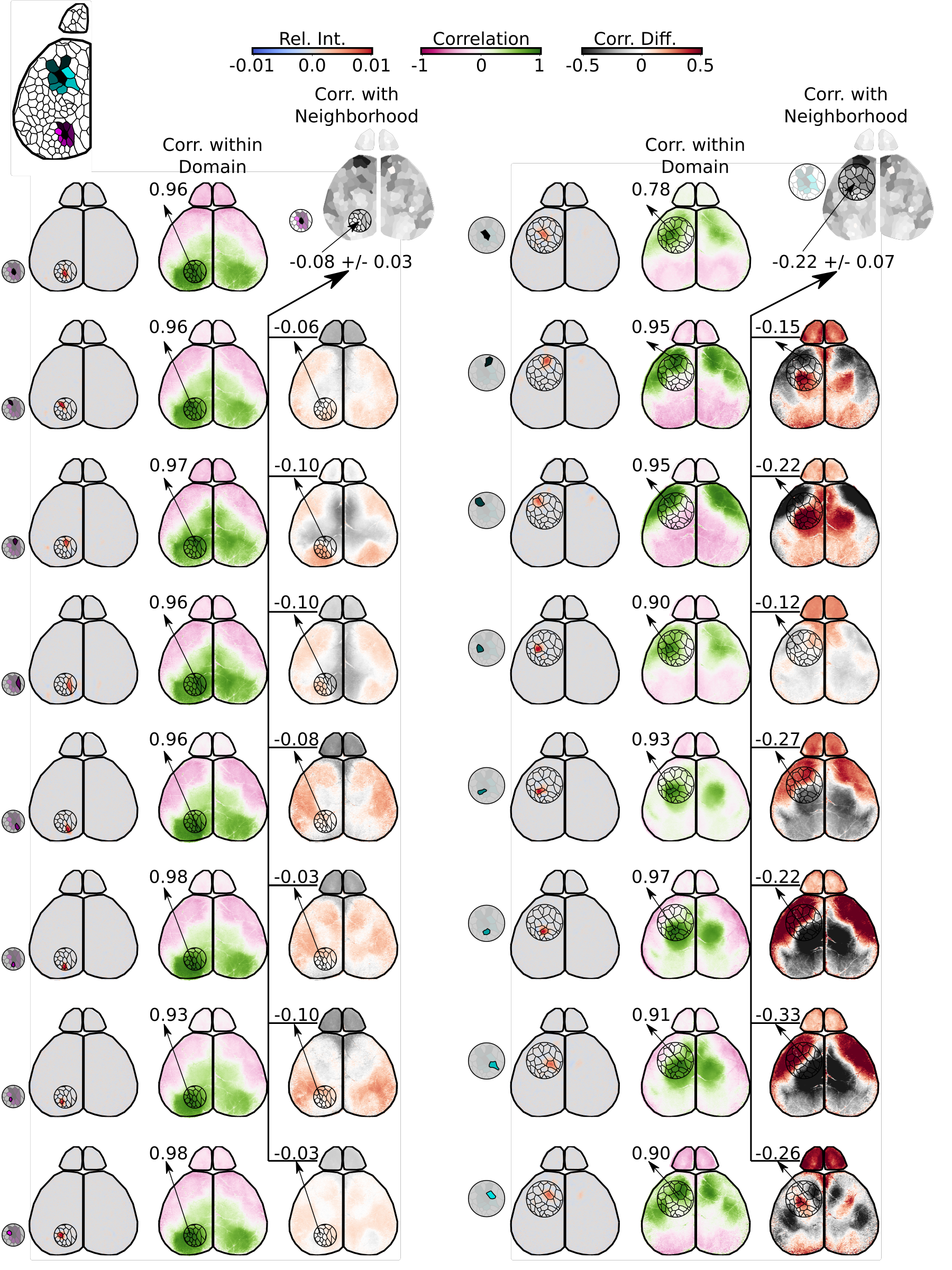

Supplement: S10 Fig — Corresponding spatial ICs matched to the timeseries domains shown in Fig 7(F). Point correlation based on the seed location of the maximal value of each IC shown in pink and green. Difference of the point correlation (gray and red) from all adjacent ICs (bottom 7E) with respect to the center domain (top domain). Values from each point correlation and difference were used in Fig 7E. (TIF) [file pcbi.1011085.s010.tif]

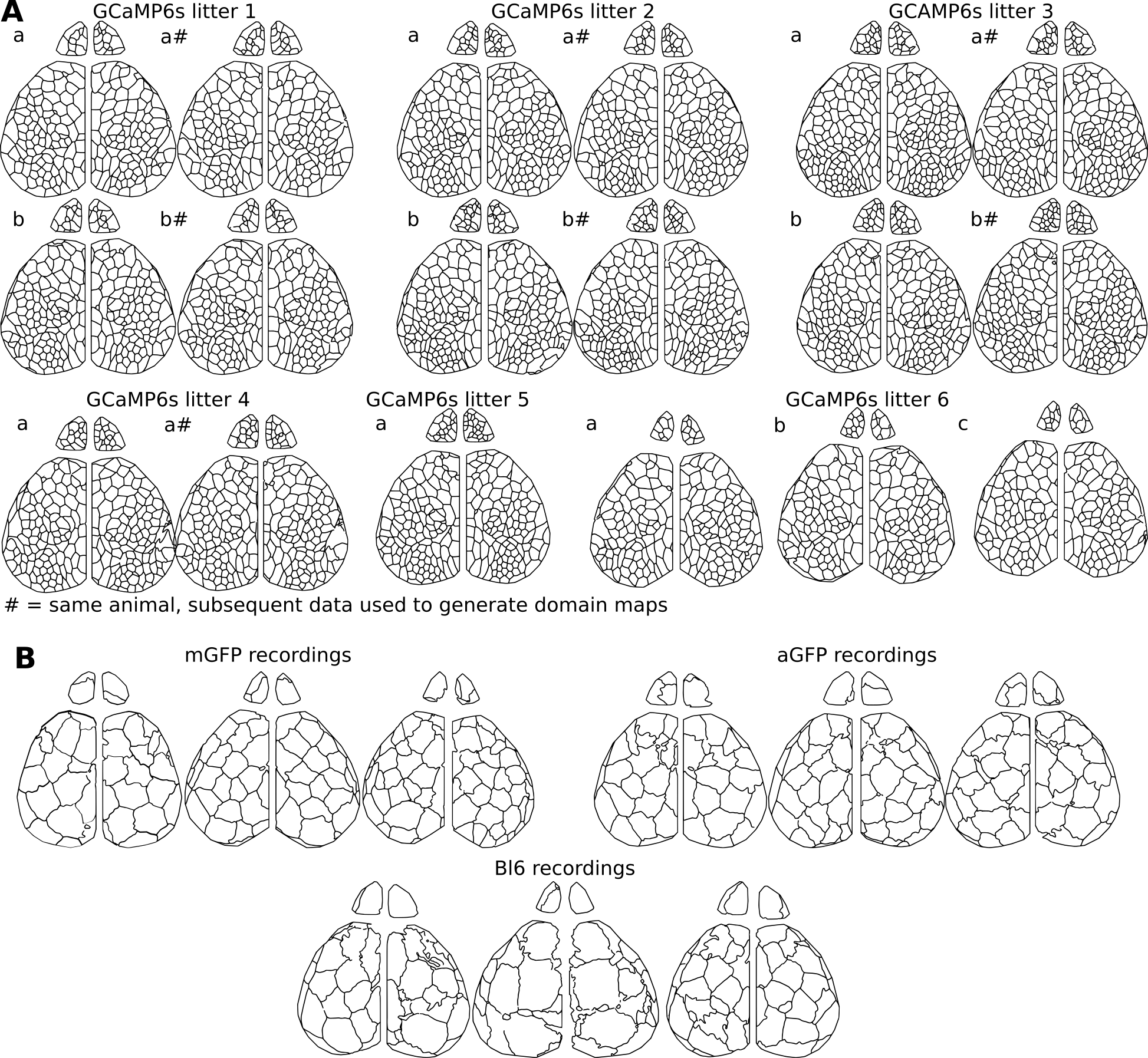

Supplement: S11 Fig — (A) Domain maps generated from Snap25 GCaMP6s recordings from littermates (a-c) and from subsequent recordings (#). (B) Domain maps generated from the three different control lines. (TIF) [file pcbi.1011085.s011.tif]

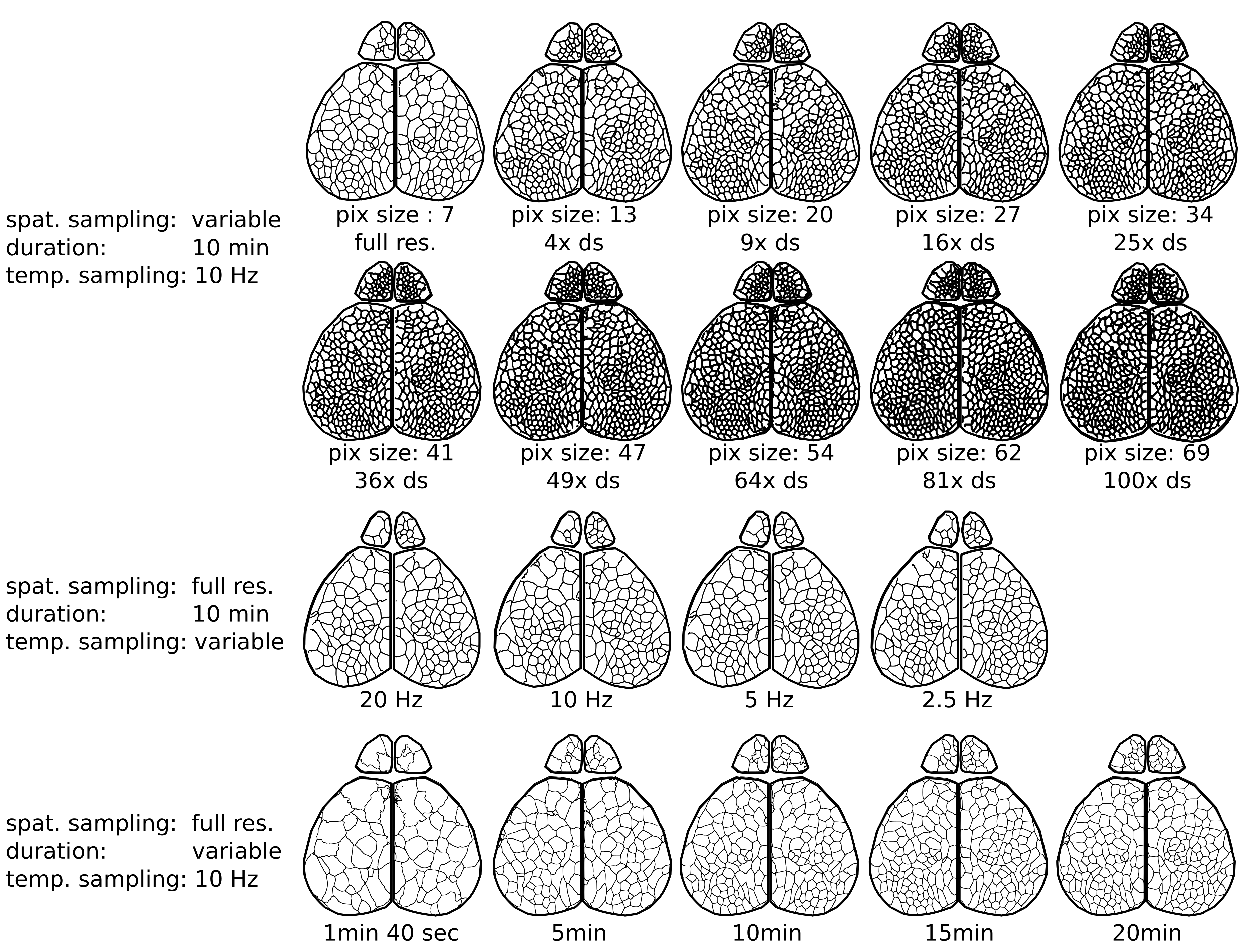

Supplement: S12 Fig — (A) Domain maps made from spatial and (B) temporal down sampling experiments. (C) Domain maps from duration experiments at 5 duration time points. (TIF) [file pcbi.1011085.s012.tif]

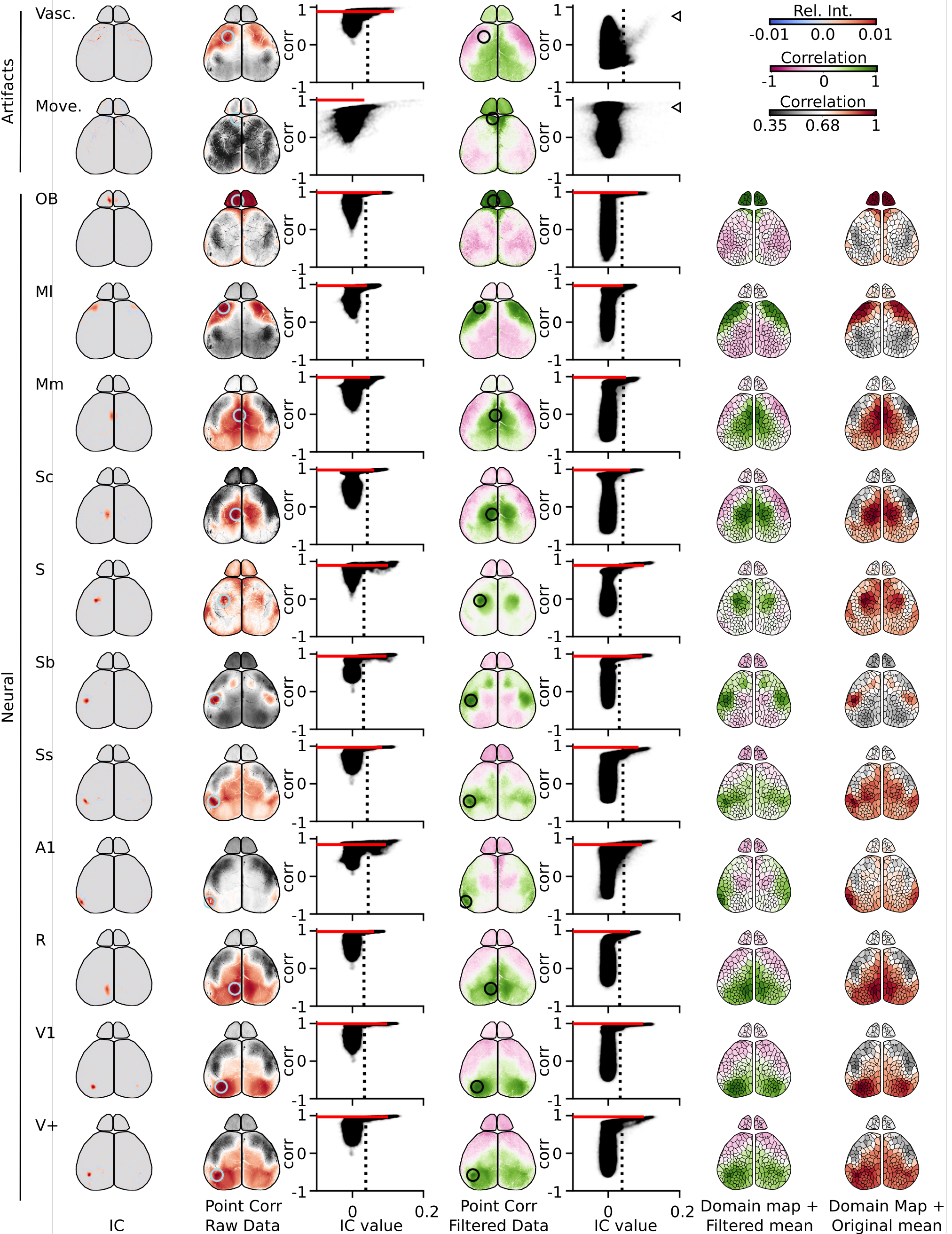

Supplement: S13 Fig — Comparison of sIC, raw data and filtered pixel wise point correlation across 2 examples of artifacts and 11 neuronal sIC, each from a distinct region of the brain. Each point correlation map was created based on the location of the maxima of the sIC (indicated by the circled regions of the point correlation maps), either using the raw data (gray to red) or the filtered data (pink to green). Pixel-wise comparison of how each correlation map plots to the IC value plotted to the right of each correlation map. Open triangles (top 2 rows, right) denote the absence of structure in the scatter plot of the artifact ICs compared to the filtered correlation map. Domain correlation maps were created by mapping each IC to which domain it most contributed, and used that domain as the seed correlation. Domain maps with gray to red colormaps have re-addition of the original mean and pink to green colormaps have re-addition of the filtered mean. (TIF) [file pcbi.1011085.s013.tif]

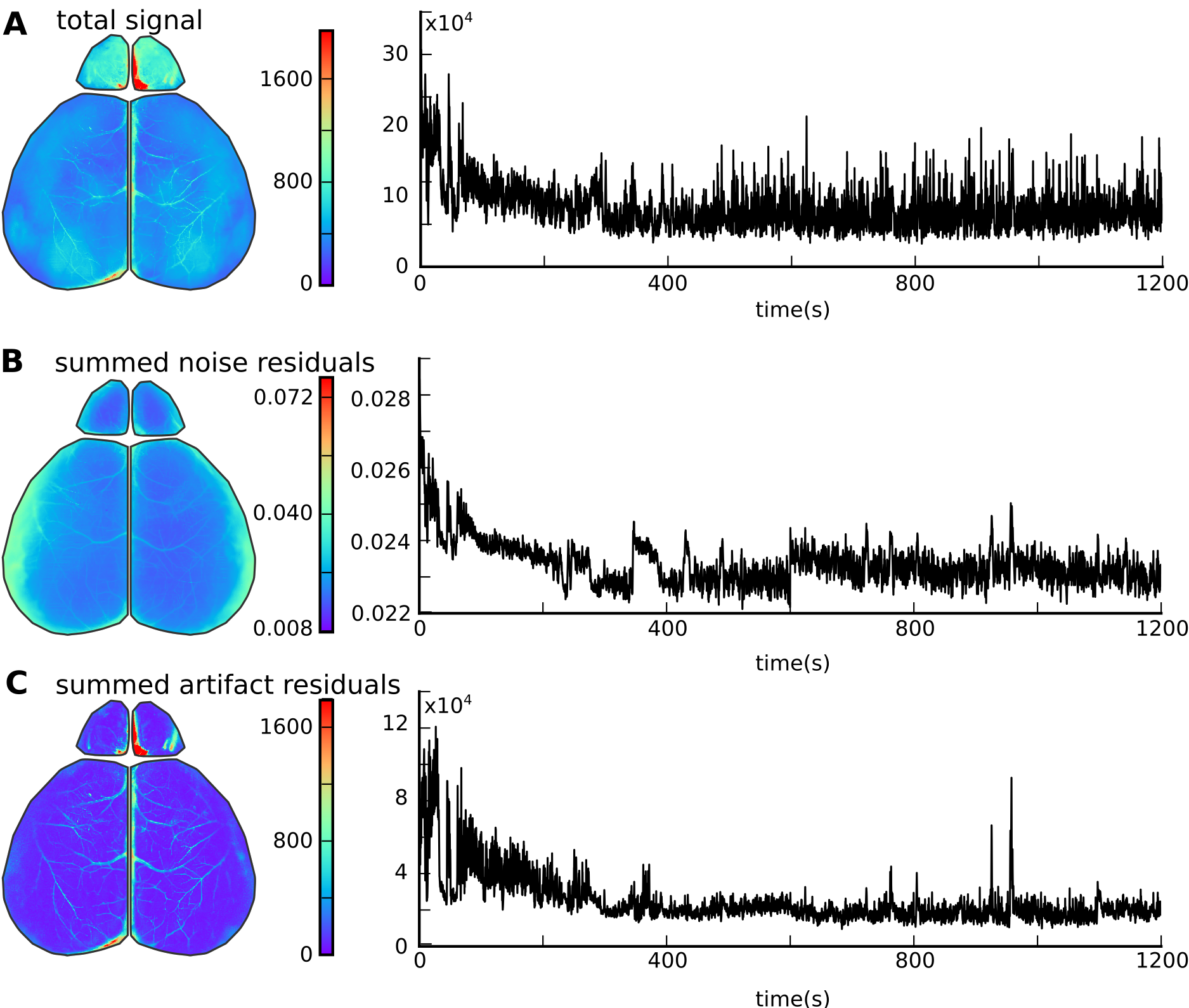

Supplement: S14 Fig — (A) The original spatial information captured as quantified by a mean subtracted absolute value projected spatially (left) or temporally (right). (B) The difference in information between the original input data and the rebuilt ICA projection, excluding noise components beyond the 25% saved in the processed file. The difference movie is projected spatially or temporally to visualize where information was lost in compression. (C) Information removed by artifact filter. The artifact movie is rebuilt and projected spatially or temporally to visualize where information was modified by the ICA-based artifact filter. (TIF) [file pcbi.1011085.s014.tif]
