# Supplementary material for: Data-driven segmentation of cortical calcium dynamics
Source: PLoS Comput Biol. 2023 May 1;19(5):e1011085. doi: 10.1371/journal.pcbi.1011085 (PMC10174627; doi:10.1371/journal.pcbi.1011085)
Supplement: Attachment — Submitted filename: Weiser_response_editors_reviewers.docx [file pcbi.1011085.s018.docx]

Dear Editors and Reviewers,

We thank the reviewers for the thoughtful comments on the manuscript. We have made four major updates in response to the reviewers and editors suggestions

1. We expanded our analysis of the effects of temporal/spatial down sampling and duration selection on how ICA behaves (Figures 2-3). Including comparable examples of spatial IC of spatial (Supp. Fig. 2) and temporal (Supp. Fig 3) down sampling; comparable examples of duration are shown in the main figure (Figure 3).  The resulting domain maps from each of the experiments were generated for comparison purposes (Supp Fig 12).
2. We have performed point correlation analysis, with points determined by the maxima of each IC, to gain a better understanding/relation of spatial IC to pixel-wise and domain correlation maps (Figure 7). We explore how each domain neighborhood relates to each other (Supp Fig 10) and discuss the assumption of independence.  Further, we show the point pixel-wise correlation of raw data, filtered data, and domain data for each region defined in the cortex (Supp Fig 13)
3. We have clarified our domain map creation and validated them using locomotion (Figures 7-10).
4. We have significantly improved the discussion section to include a more comprehensive understanding of the field, our interpretation of the results, and the limitations of our pipeline.

Both reviewers and editors have mentioned the requirement to validate proposed domain maps. We have implemented a functional validation based on locomotion (Figure 10) and clarified our position on determining regionalization (Figure 9). If this is not sufficient, we can do the cytoarchitectural registration experiment for further validation of our findings suggested by reviewer 2. We are confident that the alignment will produce similar results as the Zuang et al. 2017 paper resulting in primary cortical areas aligned centrally to our defined areas, but not well positioned along the borders due to the fluorescence scattering (Waters, 2020).  Our main interest in creating the domain maps is to segment based on unknown cytoarchitecture in the areas of the cortex that are less studied and less about the primary sensory regions. These regions are much more difficult to validate, but we hope that the domain map will allow more researchers to explore these less researched areas.

Below we respond to each comment and indicate the changes in the manuscript that address their concern.

We look forward to your response.

**# Reviewer 1**

> Calcium imaging approaches allow to investigate the neuronal activity at a wide range of spatial resolutions, from individual synapses to entire brains. Mesoscale imaging comprises the acquisition of a large portion of the brain. It is widely used in mice that express a genetic calcium indicator in neurons or projections located in superficial regions (cerebral cortex, superior and inferior colliculus). This approach allows to study, for instance, the evoked and spontaneous pattern of activity in the different cortical areas and their interactions. Because experiments are done in vivo, usually without anesthesia, the signal obtained from the movies is far from clean. Many labs obtain data using this imaging approach and usually they develop as well their own pipelines of analysis to process the images, extract data and raise conclusions.

> In this manuscript, Wieser, Mullen and colleagues provide an interesting and polished workflow for analyzing mesoscale calcium imaging data in adult mice. The method applies different strategies to increase the quality of neuronal signals and remove artifacts and noise. The method is based on a combination of principal and independent component analysis (PCA and ICA). The components are classified as neuronal, artifact or noise signal using a machine learning approach. In the manuscript, there is also an interesting exploration of the relationship between the performance of the ICA and the duration, spatial and temporal resolution of movies. In addition, the method provides a good way for filtering, compressing as well as segmenting the data. Using this segmentation and additional information, the authors construct animal-specific functional maps that are robust throughout time and litter.

> Calcium imaging analysis pipelines based on matrix factorization techniques, and based on ICA specifically, are not new in the field, as the authors state in the Discussion (lines 692 to 691). Nevertheless, one relevant point here is the code that accompanies the manuscript. The authors make a valuable contribution to the community making accessible a python toolbox, PySEAS, for the implementation of their method. The toolbox includes a well-design and friendly GUI.

> The method seems to properly identify different types of signals allowing the isolation of cleaner neuronal components. This is the main strength of the manuscript; however, I do have important questions and comments on the procedure and, also, on some statements raised by the authors. In addition, I have some major comments on how the authors build the functional maps of the cortical territory. Revealing functional maps is presented here as one of the actual applications of the method, therefore, the authors must provide arguments as convincing as possible.

**## Major points**

> 1. One of the main advantages of ICA is the dimensional reduction and filtration possibilities offered by this method, as the authors comment in the manuscript. Using ICA for making a low-dimensional representation of calcium imaging data in which artifacts have been excluded simplifies any subsequent analysis. In this case, the authors show one possible application by using the components to create functional maps of the cortex. I’m not convinced about how these maps are built but, first, I would like to raise another point. I think that the authors could try to link the independent components to physiological or biophysical concepts. For example, the independent components obtained from LFP recordings, also considered as generators of activity, have been associated to specific synaptic pathways or to different locations of the signal source (Makarov et al 2010, Herreras et al 2015). Moreover, independent components from fMRI data are related to functional networks. In other words, it would be interesting to have the opinion of the authors about why ICA is an a-priori suitable tool to analyze this type of data. In this line, it might be interesting to add some lines that justify the main assumption of statistical independence impose on the spatial dimension of the data.

We expanded our justification of utilizing ICA to explore meso-circuit dynamics in the introduction and discussion section.  We have expanded the analysis to show the relation to the proposed domain maps. We put together Figure 7 (and Supp Figure 10) to explain more clearly how the domain map is created and to explore the relationship with each spatial independent component.

> 2. The method presented in this manuscript nicely segregates calcium imaging signals increasing the quality of the neuronal component. In my opinion, this is the main achievement of the authors. To illustrate potential applications, the authors describe how the information and segmentation obtained after processing the images could be used to parcellate the cortex and build functional maps. This application would be extremely valuable for neuroscientists if the parcellation is accomplished based on the domain features or any other data-driven information. But, here, the authors manually divide the cortex in regions not only using domain features but also a reference map based on the Allen Brain atlas. Including the reference map decimates the relevance of the method for parcellating the cortex in functional regions. Actually, neuroscientists need a method to build functional maps without a reference map. As such, maps derived from control and non-canonical scenarios (development, manipulations, plasticity, pathologies, etc) could be directly and objectively compared. At the end, the reference maps should be used to validate the analysis workflow since reference maps must resemble the data-driven functional maps of the cortex derived from control mice. Is it possible to create, manually or automatically, functional maps based only in “network analysis” and “metric comparison” (line 621), or other information that does not depend on a standard reference map? If this is the case, data-driven maps should be validated analyzing to what extent they resemble already known maps. I think this is the way to reveal a convincing and relevant application of the method.

We updated our discussion of Figure 9 to clarify how we determined the regions of the domain maps.  Further, we functionally validated the map with known properties during moments of locomotion (Figure 10).

> 3. Spatial independent components are static modes and may not adequately represent the joint spatiotemporal nature of the data. In this kind of experiments, it is common to observe transient events spreading in time and space, with distinct initiation sites and wave-like flows across the cortex. I think that the static nature of spatial independent components and domain maps does not contemplate this information … is it possible to recover such information from the independent components? If not, ICA-based methods could result insufficient for those experimental questions focused on the individual properties of calcium events, especially those related with their frequency and dynamics. Could the authors discuss these observations?

The pipeline described in this paper can be used piecewise depending on the experiment and analytical requirements.  See also the discussion section relating to the applications.

> 4. Continuing with the quantification of the impact of spatial, temporal resolution and duration on ICA performance, although I appreciate the attempt for creating guiding principles for the acquisition of calcium imaging data that can be adequately filtered and disentangled using ICA, I think that there are some concepts that are not clear enough in the explanation provided by the authors. They conclude that the spatial resolution of the images is a key factor for obtaining a clear-cut separation of the different types of signals. However, as far as I know, I would say that the key factor is the number of pixels (the amount of information) and not the distance between two pixels (spatial resolution), two aspects that in their experimental design are related but not identical. In the down-sampling process for decreasing the spatial resolution the number of pixels is also reduced (even faster than the size of the pixel). By reducing the number of pixels while keeping constant the number of components, at certain point, the sample size will not be enough to properly estimate a given number of independent components (Särelä and Vigário 2003). For instance, to test if this is the case, the authors could check if spike-like components appear when the spatial resolution is low. Spike-like components are artefacts generated by ICA with insufficient sample size (Hyvärinen et al 1999). Also, down-sampling using a binning method not only changes the number of pixels but also the signal to noise ratio. If this is the case, are the results obtained at different binning factors comparable to raise conclusions only about the relationship between ICA performance and number of pixels?

We have expanded the results for our down sampling experiments (Figure 2), including comparable components between spatially (Supp fig 2) and temporally (Supp fig 3) down sampled experiments. We did find spike-like components, and included them in the supplemental figures. We expanded our discussion section to include an interpretation of these results.

It is important to note that the two labs that have produced sparse representation of sIC across the cortex heavily down sampled their data before the decomposition and were able to achieve relevant sIC (West et al. 2022; Makino et al. 2017).  While the decomposition in our hands at similar down sampling rates produces spike-like sIC in 7% of the ICs (Supp fig 2), other sIC could still hold relevant information. To achieve a representation across the cortex, we recommend as little binning as possible.

> 5. Related to the previous point, do the authors think that the performance of ICA to segregate signals depends on the signal-to-noise ratio or the bit range of the signal? Is it possible to explore these dependencies?

The signal-to-noise was improved in the temporally down sampled experiments as an increase in exposure time (summed frames in this case) will strengthen the signal and weaken the noise, independent of the spatial resolution. In the temporally down sampled experiments, we saw minimal effect on the spatial decomposition. This improved signal-to-noise ratio can be validated further in the highest down sampled experiment (2.5Hz), we see that the noise components are practically structureless, with their distribution around autocorrelation of 0; however, this was at the detriment of under sampling the signal (drop from close to autocorrelation of 1 seen in other sampling rates). See also the discussion paragraph 3.

We contemplated doing more experiments on bit range, but in the end we did not. We collected our experimental data on a 16-bit sensor, however the majority of data collected was on the scale of 12-bit.  In addition, the cMOS camera we utilized had a very low noise threshold; thus, the lower bound values were maintained low.  Our rationale for foregoing further analyses were based on two reasons. First, were based on the results of the temporal down sampling experiments. We ended up summing each of the frames independently and then normalizing by calculating the dFoF from the resulting frame (20 to 10 hz, 2x: resulting in 13-bit range; 20 to 5 hz, 4x: 14-bit range; 20 to 2.5 hz, 8x: 15-bit range). Increasing the bit-depth did not seem to change the spatial decomposition; however, we did not go down in bit-depth. Mostly due to the second reason, that the majority of scientists are no longer using 8-bit cameras and thus, no longer seemed to be a consideration of parametric selection.

> 6. Regarding the temporal resolution of the data that feed ICA, the authors conclude that it doesn't impact on the segregation of the types of signals as dramatically as spatial resolution. I would say that this is expected since the authors are running a spatial ICA. In the case of spatial ICA, each frame or image of the data is a mixture of source images that are statistically independent. In the spatial ICA model, the constraint of statistical independence is on the sources (the image vectors), while the mixing matrix or temporal courses are unconstrained. Probably, it is not relevant whether temporal courses are shorter (low temporal resolution) or longer (high temporal resolution), affecting only the number of components that can be extracted. In line with this, why does the distribution of lag-1 autocorrelations shift when time resolution is low?

See the above comment and discussion in paragraph 3.

> 7. In the Discussion, in general, the authors mainly recapitulate the Results and briefly link them to other publications. Some paragraphs, as the one starting in line 708, are just a summary of the Results. I think that more elaboration is needed to explain how the data presented here enters in the conversation with the published literature of the field. Just one example, in the Author Summary, they mention that the signal from neurons from different depths is a confounding factor in this type of experiments. I think that the method presented in this manuscript does not get rid of this problem. However, the authors do not discuss further this particular issue, and general limitations of the technique are vaguely mentioned in lines 705-706. Please, elaborate on knowledge/technical gaps that have been filled, applications, limitations of the method, etc.

We have updated the discussion section regarding these points.

**## Minor comments**

We addressed the following minor comments in the manuscript.

> 1. I’m not sure if the term eigenvector is correct in this case. I’m not sure if independent components are proper eigenvectors, as it is the case for principal components analysis where components are the eigenvectors of the cov-matrix of the data. Is this correct?

> 2. In line 182, the authors mention PC (Principal Component) when in fact they mean “independent component”. And I think that this mistake appears also in Figure Supplementary 1, where they label the different panels as Principal Component Analysis: “PCA viewer”, “Select principal component analysis”. Please, clarify these issues.

> 3. The use of terms “noise” and “artifact” is sometimes confusing, as in lines 256-261 and related figures. Please, revise the whole text on this regard.

> 4. In line 100, bibliographic reference is incorrect, 23 instead of 22.

> 5. In Figure 1, caption mentions a dashed line in panel A that is not present or visible.

> 6. In line 319, the omega symbol is missing.

> 7. In line 78-79, “NNNxNNN” pixels is not the spatial resolution.

> 8. In Figure 1C and others, the scale of Relative Intensity is not the same in the brain schemes and in the traces.

> 9. In line 205, please rephrase “High resolution spontaneous activity”.

> 10. In Figure 2D, mean artefact curve does not seem to level off.

Now Figure 3A

> 11. In line 320, replace purple by blue and panel letters seem to be missing or wrong in Figure Supplementary 2.

Now supplementary figure 4

> 12. Please, revise the references in the text for Figure 5F, Supplementary 5A and C, and Supplementary 6C.

Now Supplementary Figures 7 and 8

> 13. I think that the caption of Figures Supplementary 6 and 7 are swapped.

Now Supplementary Figures 8 and 9

> 14. In general, in the figures showing neural independent components (Figures 1C, 3A, and Supplementary 1) there are negativities (in blue) surrounding the positive part of the component (in red). Which is the origin and interpretation of these negative values?

The negative values are reciprocal activations to the positive values. A few interpretations based on the type of sIC:

There is a high prevalence of sIC with negative values in movement artifacts. As the surface of the brain moves during the recording, along the blood vessels the light refraction changes will increase signal to where the blood vessel was located, but intensity will decrease to where the blood vessel is newly located (see Figure 4A; first other artifact component: o1).

In the cases of the neural components, we primarily see slight negative values surrounding the main neural sIC. These are not as intense and are less reliable. We interpret these weak negative values representatives of the waveform flowing across the region, when that main region is activated the immediately surrounding tissue is less likely to be active (Figure 4A).

In the few cases where the neural sIC have stronger, structured negative values, we observe these in neural sIC with low variance (see Figure 7C) and we interpret these sIC as modulators of other neural sIC. These neural activations usually do not have influence on the domain maps, but are used during the reconstruction of the biological signal (an argument to do analyses on the reconstructed data rather than each sIC).

> 15. The sixth paragraph of the discussion is hard to read and understand.

**# Reviewer 2**

> The authors provide potentially novel work using a higher resolution form of typical mesoscale wide field imaging coupled with analysis pipelines to recover specific networks. The work includes using 7 micrometer pixels but still employing a relatively standard low resolution mesoscale wide field imaging setup. The images undergo extensive processing using an independent component analysis (ICA) pipeline to break up time series images into components elements. The ICA procedure is done for a snap 25 gcamp mouse as well as several different control line mice that express gfp in astrocytes or microglia. The control lines expressing gfp only are put forward as a means of parsing out signal versus artifacts due to pixel noise, vessels or brain movement. Overall the findings are surprising in that the widefield images can be decomposed into many more sub-image domains that were then were previously reported with other methods including seq nmf (Buschman lab). The sub regions are then clustered into large groupings that tend to be consistent with large scale anatomical relations.

> The work would be significant and novel if the small sub-regions that are identified can be verified to overlap with known functional activations and confirmed in some way to be units on their own rather than some by-product of the analysis.

We updated and expanded our discussion of Figure 9 for a complete picture of how we determined the regions of the domain maps.  Further, we functionally validated the map with known properties during moments of locomotion (Figure 10).

**## Points**

> Previous work on the resolution of wide field imaging suggests resolution that is likely insufficient to resolve the small clusters that are observed. https://elifesciences.org/articles/59841 see Fig 2 how do the small activations compare with this expected resolution?

The strength of this segmentation from the ICA algorithm is due to the increased number of activations (see figure 3), to determine independent mixtures of signals. We have expanded our analysis and discussion on our interpretation of each component and the limitations of this approach.

> Figure 1 examines potential motifs extracted using the ICA procedure. Surprisingly, high resolution (small ½ width) and small areas thought to be neuronal components are shown in panel c and other components shown to be artifacts are also indicated. It would be important to show that these small discrete areas thought to reflect real signals are both within the resolution of wide field imaging and that these areas also represent independently functioning cortical areas if time plots from different roi's are shown. They could also do something like a seed pixel local correlation analysis again to provide information that these areas are indeed independent elements as they proposed. Thus far the evidence that these are unique regions with independent function is not convincing.

We performed a seed correlation analysis based on each independent component (see Figure 7, Supp fig 10, Supp fig 13).  We showed the relation of each sIC, its corresponding domain, and its derived seed correlation map. Further, we showed evidence that each domain was distinct in correlation, even within a domain neighborhood by looking at the subtraction of the two seed correlation maps.  We show that each sIC has a distinct association based on correlations across the cortex, and not just in a local manner.  As a reminder, uncorrelatedness is only partially independent (Hyvärinen et al 1999), and as such, the direct relationship between sIC and the point correlation map show that highly correlated regions are distinct (Figure 7C, Supp fig 13).  The independence is based on all pixels and not just local neighborhoods.

Out of this analysis, we see that many of the primary sensing units cortex (and olfactory bulb, as you have already point out) are less different than their respective neighborhood, and motor and higher order cortical domain are more different than their respective neighbors.

> Figure 2 shows the variance of the eigenvector's and is used to argue that neural signals are indeed separated from artifacts based on their autocorrelation and their variance profiles. However, to do this one needs to assume that artifacts and neural activity operate on significantly different time scales and spatial properties. In the case of vascular noise this could potentially be on a slow second time scale similar to neuronal activity or breathing or large body movements.

We have clarified the writing to address this concern. In the field there is a distinction of structured noise vs unstructured noise.  In this paper, we have called all structured noise as artifact. All unstructured noise as noise. The autocorrelation and variance were used to distinguish neuronal and artifact sIC from unstructured noise. The original submission was unclear and the interpretation could have been reached that the artifacts are noise. We have corrected the writing to reflect otherwise.

> Figure 3. Demonstrates the recovery of potential activity like motifs within gcamp expressing mice and control gfp lines. Surprisingly there is a large number of neuronal like activations within the control gfp expressing lines in figure 3b. It is also unclear that these small potential neuronal activations are mapped to known sensory or neural anatomical motifs again bringing up the concern about functional resolution in vivo and spreading of signal which is known to occur (first para concern too). different control lines were employed, it is not clear that the fluorescence properties of all the lines is indeed comparable. If the glio mice have a poorer signal to noise ratio it could potentially recover more artifacts from them than a brightly expressing neuronal line. Ideally the gfp control lines should be better matched to the neuronal signal expected lines (snap25) in terms of laminar expression profile (layer specific) and also absolute intensity and SNR properties. This could be accomplished using a similar neuronal expressing line for gfp alone or some controls to show that these are somehow comparable.

Unfortunately, when we began looking for the snap25 expressed GFP lines they were not available. Even if we were to get the snap25 GFP lines, the base fluorescence is drastically different from GCaMP lines and the fluorescence properties and are not comparable. To account for the difference, we changed the light intensity to try to match baseline fluorescence between the GCaMP and GFP lines.

The GFP from a single source, much like the GCaMP, will scatter from an individual cell out the 200um (Waters, 2020), well past the single cell resolution. Both the microglia and astrocyte were selected because of the equidistant tiling effect across all cortical areas, similar to what we would expect from Snap25 GFP mouse lines. It is difficult to imagine a case where GFP from adjacent cells, that are also equally distributed across regions, would produce dramatically different results. In fact, we saw very similar results from two different cell groups, astrocytes and microglia (see Supp Fig 6). We expect we would see the same from neuronal GFP lines as well. Further, the domains isolated from the GCaMP expressing mice are VERY different from our control animals and we present strong evidence that it is not because of differences in cellular expression but that of GCaMP.

The neural-like components are regionalized based on arterial patterning and not based on functional domains.  A direct connection to the hemodynamic effect that can be seen with the sustained contrasting agent (constant GFP). Whereas with GCaMP, we do not get this same separation due to the dynamic, non-sustained contrast.  We still get other vascular components (most likely venous) from both GFP lines that separate independently from any tissue changes.

> Figure 4 shows the distribution of recovered activations within the different lines of mice. The figure is notable in that the artifact regions nicely aligned with the expected midline. there are some assumptions about the time course of vascular components across mouse lines. Here the different gfp lines should be potentially measuring the same thing but the time courses can be quite different since they are different cell populations and compartments.

See the comment above

> Figure 6 the summing of the different components is intriguing but the authors again need to show that the components exhibit some expected independence by showing time plots from additional adjacent regions. One could also imagine examining some correlational structure for doing some functional mapping such as retinotopic using methods that have previously been employed including the work of Waters (elife) and others to map subdomains.

We would love to get our hands on some non-resting state data to see how ICA behaves. We are a developmental neuroscience lab and thus have focused on spontaneous activity. We believe that if ICA was done on behavioral data, it would bias the segmentation to the trained/tested state (see discussion section, paragraph 6).

Please refer to Figures 7 and 9. Supp figures 10, 13.  This is the suggestion of cytoarchitecture mentioned above.

> Figure 7 is welcome in that the maps seem to resemble what is expected for large-scale functional and anatomical relationships in the cortex that have been previously reported. However many of the intra cortical correlations raise some issues such as if the olfactory bulb regional correlation is very high how could one pull out separate areas? There could also be a bit better description for how the small regions are inserted into the macro clusters?

This is now Figure 9 and 10.  We updated Figure 7 to address the small regions inserted into the macro clusters. In short, the small domains are typically the result of secondary region(s) from a single multiregional sIC (sIC with bilateral symmetry or multiple intracortical regions), out-competing other sIC.

In terms of the olfactory bulb, we have debated these results extensively.  We have always been on the fence about what it means and if we should include them in our analysis. A few continuing facts that we noticed: 1) The skull is thicker above the OB than the rest of the cortex, therefore more fluorescence dispersion.  2) The signal is fainter, so much that many times the signal does not pass our wavelet analysis red-noise threshold. 3) OB has MANY vascular components surrounding it (sagittal sinus and rostral rhinal vein being the two largest) 4) The skull sutures are prone to residual membranes and bleeding artifacts. We do get odd sIC from the OB and it is more difficult to distinguish artifacts from neural components.  Our pipeline is optimized for the cortex and feel confident about those results.  In the end we kept the OB in the analysis, since we have seen that they still correlate well with locomotion (which is expected) and still emit meaningful signal.

> Panel f would be nice to have better color coding of the specific areas and there should be more discussion about the significance of these results. It is not necessarily surprising that an individual animal would be more similar to itself than another mouse because of issues of rising with the alignment or quality of the window preparation. How much of this is animal specific brain activity versus the conditions around the imaging of different mice?

We have now updated panel F (now Figure 9E) to include color to each region.

The difficulty of comparing two maps of multiple regions, across two hemispheres should not be understated. We attempted to reduce the experimental variation by independently adjusting the coordinate position of each hemisphere in order to align all 10 regions such that it maximizes the jaccard index (ie. that minimizes the positional variation).  However, we did not adjust the tilt of the map or the rotational angle due to the computational difficulty of transformation of the map.  We cannot outright exclude the possibility of experimental variation, but we have attempted to minimize its influence (see methods section).  Further, we looked at the map as a whole and not piecewise. It is possible that certain regions are more similar than others, but collectively in the map they would not be.
